# Posttranslational modifications of serine protease TMPRSS13 regulate zymogen activation, proteolytic activity, and cell surface localization

**DOI:** 10.1016/j.jbc.2021.101227

**Published:** 2021-09-22

**Authors:** Carly E. Martin, Andrew S. Murray, Kimberley E. Sala-Hamrick, Jacob R. Mackinder, Evan C. Harrison, Joseph G. Lundgren, Fausto A. Varela, Karin List

**Affiliations:** 1 Department of Pharmacology, Wayne State University, Detroit, Michigan, USA; 2 Department of Oncology, Wayne State University, Detroit, Michigan, USA; 3 Division of Hematological Malignancies and Cellular Therapy, Duke University, Durham, North Carolina, USA; 4 Department of Biochemistry and Molecular Biology, University of Kansas Medical Center, Kansas City, Kansas, USA

**Keywords:** serine protease, N-linked glycosylation, phosphorylation, cell surface protein, protease inhibitor, type II transmembrane serine protease, TTSP, TMPRSS13, HAI-2, ER, endoplasmic reticulum, ERAD, ER-associated degradation system, GPI, glycophosphatidylinositol, HAI, hepatocyte growth factor activator inhibitor, HGF, hepatocyte growth factor, HMW, high molecular weight, KLK, kallikrein-related peptidase, L, lipoprotein receptor class A, MSPL, mosaic serine protease large-form, PCSK6, proprotein convertase subtilisin/kexin-6, PI-PLC, phosphatidylinositol-specific phospholipase C, PNGase F, peptide:N-glycosidase F, SP, serine protease, SRCR, scavenger receptor cysteine rich, TM, transmembrane, TTSP, type II transmembrane serine protease

## Abstract

TMPRSS13, a member of the type II transmembrane serine protease (TTSP) family, harbors four N-linked glycosylation sites in its extracellular domain. Two of the glycosylated residues are located in the scavenger receptor cysteine-rich (SRCR) protein domain, while the remaining two sites are in the catalytic serine protease (SP) domain. In this study, we examined the role of N-linked glycosylation in the proteolytic activity, autoactivation, and cellular localization of TMPRSS13. Individual and combinatory site-directed mutagenesis of the glycosylated asparagine residues indicated that glycosylation of the SP domain is critical for TMPRSS13 autoactivation and catalytic activity toward one of its protein substrates, the prostasin zymogen. Additionally, SP domain glycosylation-deficient TMPRSS13 displayed impaired trafficking of TMPRSS13 to the cell surface, which correlated with increased retention in the endoplasmic reticulum. Importantly, we showed that N-linked glycosylation was a critical determinant for subsequent phosphorylation of endogenous TMPRSS13. Taken together, we conclude that glycosylation plays an important role in regulating TMPRSS13 activation and activity, phosphorylation, and cell surface localization.

The type II transmembrane serine proteases (TTSPs) are a family of 17 cell surface-anchored proteases, divided into four different subfamilies: HAT/DESC, hepsin/TMPRSS, matriptase, and corin (1, 2, 3, 4). TTSPs are synthesized as inactive zymogens that require cleavage at an arginine or lysine residue for activation, and, upon zymogen activation, the catalytic serine protease (SP) domain remains tethered to the remainder of the protease by a disulfide bridge (1, 5). The dysregulation of many TTSP family members has been implicated in the development and progression of various cancers (6, 7, 8, 9, 10, 11, 12, 13, 14, 15, 16, 17, 18, 19, 20, 21, 22, 23, 24). Transmembrane Protease, Serine 13 (TMPRSS13, also known as Mosaic Serine Protease Large-Form (MSPL)), a TTSP that belongs to the hepsin/TMPRSS subfamily, has recently been implicated as a pro-oncogenic protease in both breast and colorectal cancers (25, 26).

TMPRSS13 is distinct from the rest of the TTSPs due to its long, intrinsically disordered and highly phosphorylated intracellular domain (1, 27). TMPRSS13 was first cloned from human lung in 2001 (28), and since then its expression has been identified on epithelial cells in various tissues, such as the epidermis and respiratory epithelia (28, 29, 30, 31, 32); TMPRSS13-deficient neonatal mice exhibit mild epidermal barrier defects that subside as they reach adulthood (29). TMPRSS13 is able to cleave and activate pro-hepatocyte growth factor (HGF) *in vitro* (33). In cancer, TMPRSS13 plays a promotional role by supporting primary tumor growth and metastasis *in vivo* (26). Furthermore, TMPRSS13 promotes cell survival, invasion, and resistance to drug-induced apoptosis in cancer cells (25, 26). TMPRSS13 also plays a role in cleavage of viral hemagglutinin to increase pathogenicity of influenza viruses (34, 35), and it cleaves and activates SARS-CoV-2 spike protein to promote viral entry and replication (36, 37, 38). Therefore, TMPRSS13 has emerged as a novel target for the design and discovery of drugs for treating cancer and viral infections. At the biochemical and cellular levels, however, TMPRSS13 has not been extensively characterized. We have previously shown that TMPRSS13 is modified by glycosylation (27), but the functional role of this posttranslational modification has not been determined. Glycosylation can affect catalytic activity and the recognition, specificity, and binding affinity of substrates/inhibitors, which are important parameters to consider for drug development strategies. To determine the role of glycosylation for TMPRSS13 function, we performed a comprehensive study using multiple biochemical and cellular approaches.

Asparagine (N)-linked protein glycosylation is a process that entails the stepwise addition of carbohydrates onto a lipid molecule and the subsequent transfer of the oligosaccharide group onto an asparagine residue of a newly formed protein in the endoplasmic reticulum (ER) (39, 40). The consensus sequence motif for N-linked glycosylation is N-X-S/T, where X is any non-proline amino acid (39). N-linked glycans serve various functional roles, including assisting in proper polypeptide folding, protein quality control, and protein solubility (39, 41, 42). Several TTSP family members are known to be glycosylated, including hepsin (43, 44), enteropeptidase (45, 46), matriptase (47, 48), matriptase-2 (49), corin (42, 46, 50, 51), TMPRSS11a (52), and TMPRSS3 (53), where glycosylation is important for one or more processes including zymogen activation, catalytic activity, stability, trafficking to the cell surface, and shedding. The function of posttranslational modifications for individual proteases, while frequently modulating similar processes, cannot be predicted since there is currently no known general pattern, and glycosylation can have differential effects on catalytic activity and cellular properties.

This study is the first to investigate the role of N-linked glycosylation for the autoactivation, proteolytic activity, cellular localization, and phosphorylation of TMPRSS13.

## Results

### N-linked glycosylation of the serine protease domain is critical for TMPRSS13 autoactivation

**Figure 1:**
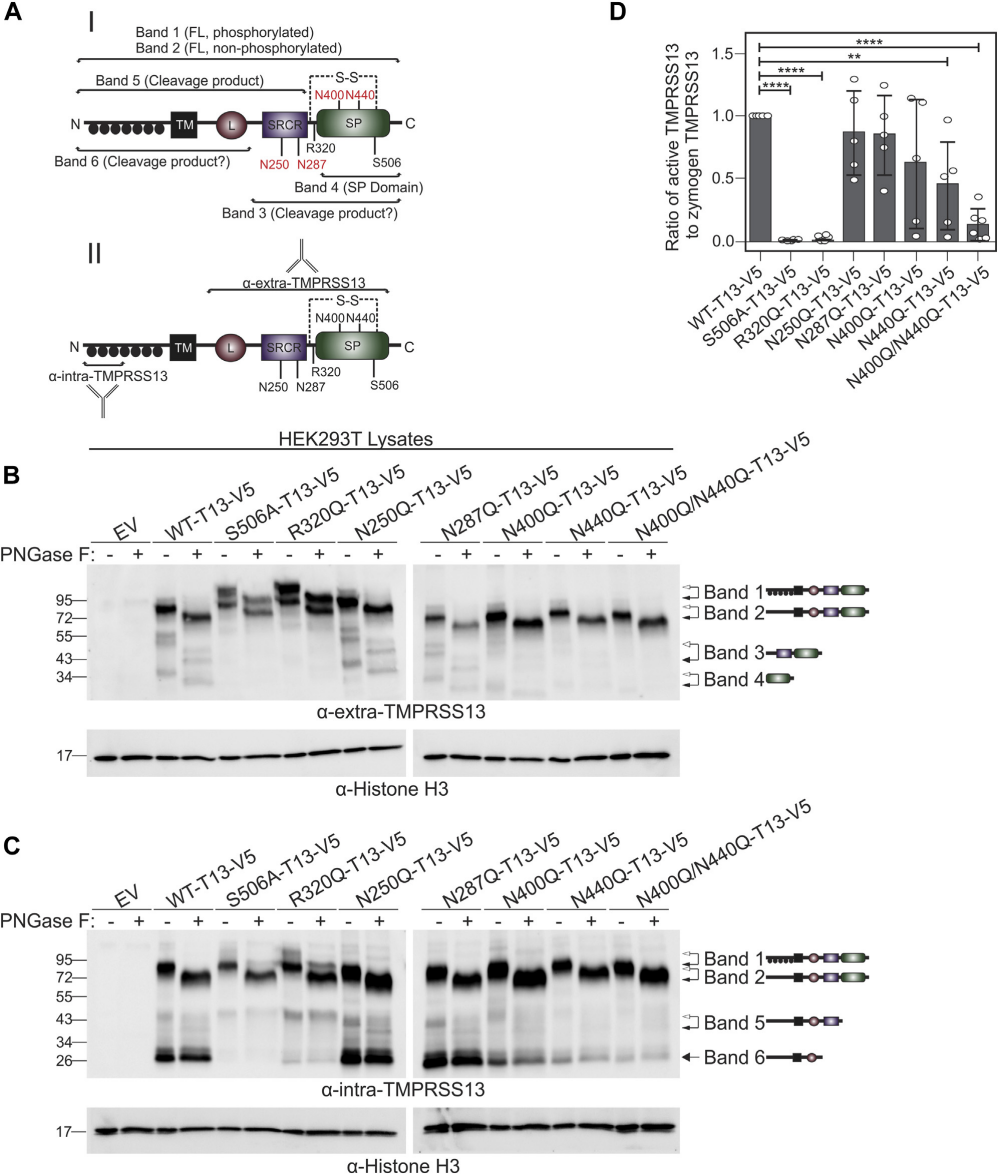
**N-linked glycosylation of TMPRSS13 is important for autoactivation.***A*, schematic representation of TMPRSS13 showing full-length TMPRSS13 and cleavage fragments (I) and epitopes for anti-intra-TMPRSS13 and anti-extra-TMPRSS13 antibodies (II). The activation cleavage site is located at R320, the catalytic serine residue is located at S506, and the four sites of N-linked glycosylation are N250, N287, N400, and N440 (in *red text*). The disulfide bridge tethering the SP domain to the stem region is denoted with “S-S” and a *dashed line*. Phosphorylation of the intracellular domain is indicated by *filled black circles*. *B* and *C*, HEK293T cells were transfected with TMPRSS13 constructs and proteins from cell lysates were separated by SDS-PAGE under reducing conditions using 10% gels and analyzed by western blotting. Lanes with protein extracts treated with PNGase F prior to SDS-PAGE are indicated by “+” and those that received no PNGase F are indicated by “−”. Proteins were detected using anti-extra-TMPRSS13 (*B*), anti-intra-TMPRSS13 (*C*), or anti-histone H3 antibodies. *Arrows* to the *right* of the western blots indicate TMPRSS13 bands defined in *A* (*white arrowheads*; TMPRSS13 form prior to PNGase F treatment) and their representative schematics (*black arrowheads*; TMPRSS13 forms after PNGase F treatment). *D*, bar graph showing the ratio of the released SP domain to the full-length TMPRSS13 protein, normalized to WT-TMPRSS13. Error bars are representative of standard deviation. One-way ANOVA with Dunnett’s multiple comparisons post-hoc test was used to determine significance of SP-domain release compared with WT-TMPRSS13. Results for at least five biological replicates are shown. ∗∗*p* ≤ 0.01, ∗∗∗∗*p* ≤ 0.0001. FL, full-length TMPRSS13; L, lipoprotein receptor class A domain; SP, serine protease domain; SRCR, group A scavenger receptor cysteine-rich domain; TM, transmembrane domain.

TMPRSS13 has four predicted sites of N-linked glycosylation in its extracellular domain, based on both the N-X-S/T consensus sequence and the surrounding amino acid context (27, 54). To investigate the role of N-linked glycosylation for TMPRSS13 functions, site-directed mutagenesis was performed to selectively mutate these four putatively glycosylated asparagine residues (N250, N287, N400, N440) into glutamine residues. N250 and N287 are both located in the scavenger receptor cysteine rich (SRCR) region of TMPRSS13, while N400 and N440 are located in the SP domain (Fig. 1*A*). The lipoprotein receptor class A domain (L) does not contain predicted glycosylation sites. The resulting plasmids encode the full-length V5-tagged human TMPRSS13 variants; N250Q-T13-V5, N287Q-T13-V5, N400Q-T13-V5, N440Q-T13-V5, and N400Q/N440Q-T13-V5, the latter of which has both N-glycosylation sites in the SP domain mutated.

HEK293T cells were transfected with wild-type (WT) TMPRSS13, S506A (catalytically dead; the serine residue in the catalytic triad is mutated to alanine (27)) TMPRSS13, R320Q (zymogen locked; the zymogen activation site is mutated to glutamine (27)) TMPRSS13, and the five N-linked glycosylation mutant plasmids. As described previously, WT-TMPRSS13 is capable of autoactivation by zymogen cleavage at R320, which releases its proteolytically active SP domain under reducing SDS-PAGE conditions (27). S506A and R320Q TMPRSS13 mutants serve as negative controls for proteolytic activity and activation/autoactivation, respectively (27). Forty-eight hours after transfection, cells were lysed and treated with peptide:N-glycosidase F (PNGase F), an enzyme that removes most types of N-linked glycosylation including high mannose, hybrid, and complex oligosaccharides (55, 56, 57).

Whole cell lysates with or without PNGase F treatment were separated by SDS-PAGE under reducing conditions, which disrupts the disulfide bond that tethers the SP domain to the stem region of TMPRSS13, thereby allowing visualization of the released active SP domain by western blotting. Western blots were probed with two antibodies directed against different epitopes of TMPRSS13: one that recognizes amino acids 195 to 562 on the extracellular portion of the protease (α-extra-TMPRSS13) and one that recognizes the first 60 amino acids of the protease (α-intra-TMPRSS13); antigens are indicated in Figure 1*A*, II.

Detection of WT-TMPRSS13 using the α-extra-TMPRSS13 antibody (Fig. 1*B* and Fig. S1) shows that full-length nonphosphorylated TMPRSS13 migrates in accordance with its predicted molecular weight of ∼61 kDa plus the addition of a ∼5 kDa V5 tag (∼66 kDa total) upon treatment with PNGase F compared with the glycosylated form of TMPRSS13 at ∼80 kDa without PNGase F treatment (**Band #2**) indicating that PNGase F removes N-linked glycans from full-length TMPRSS13. The released SP domain migrates as a ∼36 kDa band without PNGase F treatment (**Band #4**), representing the catalytically active protease domain, as we have previously demonstrated by α2-macroglobulin capture (27). This band also migrates as a lower-molecular-weight species upon PNGase F treatment, further confirming that the SP domain is glycosylated.

Notably, we have shown that the S506A and R320Q mutants are highly phosphorylated in their intracellular domains and display a phosphorylated high-molecular-weight (HMW) form by western blotting, and phosphorylation of WT-TMPRSS13 is also observed upon coexpression with the cognate inhibitor hepatocyte growth factor activator inhibitor (HAI)-2 (27). For the S506A and R320Q mutants, the HMW band ∼95 kDa (**Band #1**) is detected, indicative of phosphorylation of the intracellular domain (as detailed in (27)). The phosphorylated form shifts downward upon PNGase F treatment indicating that this form, like nonphosphorylated full-length TMPRSSS13, is also glycosylated in agreement with our previously published observations (27). The S506A and R320Q mutants are rendered enzymatically inactive or are zymogen activation deficient, respectively; no autoactivation leading to released SP domain is detected in either mutant.

In the N250Q and N287Q single mutants, the released SP domain is readily detected, suggesting that abrogating glycosylation in the SRCR domain does not significantly impede the ability of TMPRSS13 to autoactivate (quantitation of released SP domain relative to full-length TMPRSS13 for all constructs is shown in Fig. 1*D*). However, there is a reduced ratio of the SP domain compared with the full-length TMPRSS13 protein in the N440Q single mutant, indicative of a role for this site in its autoactivation (Fig. 1*D*). The N400Q/N440Q double mutant is detected as a full-length, nonphosphorylated protein with little to no detectable release of its SP domain. Thus, the mutation of both glycosylation sites in the SP domain of TMPRSS13 significantly decreases activation of the TMPRSS13 protein, indicating that N-linked glycosylation at both sites in the SP domain is critical for efficient autoactivation (Fig. 1*D*).

WT, N250Q, and N287Q TMPRSS13 also display a ∼55 kDa band (**Band #3**) that, based on its molecular weight and the fact that it shifts to a lower-molecular-weight form upon treatment with PNGase F, may represent a cleavage product resulting from a cleavage site between the transmembrane (TM) and SRCR domains (**Band #3**). Since this form is not observed in the S506A and R320Q mutants, and it is detected at reduced levels in the N400Q/N440Q single and double mutants, it may represent an autocleavage fragment (see Fig. 1*C*, Band #6 for detection of the potential other half of cleaved TMPRSS13 using the intracellular TMPRSS13 antibody). Additionally, it is noteworthy that while the N400Q/N440Q double mutant has a significant reduction in autoactivation, similar to S506A and R320Q, it does not show a detectable phosphorylated form. This implies that phosphorylation of TMPRSS13 is not solely a consequence of reduced catalytic activity of the protease.

When parallel western blots were probed with the α-intra-TMPRSS13 antibody that is directed against the intracellular portion of TMPRSS13, the full-length nonphosphorylated TMPRSS13 and HMW phosphorylated forms are detected similarly to the α-extra-TMPRSS13 blot (Fig. 1*C* and Fig. S1) with a shift to lower-molecular-weight forms upon PNGase F treatment. Two additional forms are visible in WT-TMPRSS13: one at ∼43 kDa (**Band #5**) and one at ∼26 kDa (**Band #6**). Based on the molecular weight of Band #5, and because it shifts to a lower-molecular-weight form upon PNGase F treatment, this form likely represents the intracellular portion of TMPRSS13 following cleavage at R320, the canonical activation site. We hypothesize that Band #6 represents the intracellular fragment of TMPRSS13 following cleavage at an additional cleavage site (extracellular fragment, **Band #3** in Fig. 1*B*) between the transmembrane (TM)-domain and SRCR domain. This prediction is based on its molecular weight and the observation that it does not shift downward upon PNGase F treatment, indicating that it represents a cleaved form without glycosylation sites (fragment including the intracellular domain, the TM domain, and potentially the L domain, see Fig. 1*A*). As Band #6 is not detected in the S506A mutant, and is observed at reduced levels in the R320Q mutant, it is plausible that this cleavage product is generated by TMPRSS13 autocleavage as mentioned above. In comparison to WT-TMPRSS13, N400Q, and N440Q single mutants show greatly reduced levels of Band #5 and Band #6, and the level is further reduced in the N400Q/N440Q double mutant suggesting that mutations of N-glycosylation sites in the SP domain impair the proposed autocleavage of TMPRSS13.

To confirm that the western blot results using HEK293T cells were reproducible in a different cell type, Cos7 cells were transfected with WT-TMPRSS13-V5, S506A-TMPRSS13-V5, and N400Q/N440Q-TMPRSS13-V5. Cell lysates were analyzed by SDS-PAGE and western blotting using the α-extra-TMPRSS13 and α-intra-TMPRSS13 antibodies for detection as described above in HEK293T cells, and the observations were reproducible in the Cos7 cell line (Fig. S2*A*). Importantly, the addition of the V5-tag does not appear to alter the properties of WT-TMPRSS13 and mutated forms regarding autocatalytic ability/SP domain release, or phosphorylation status as shown by transfection of HEK293T cells with untagged WT-TMPRSS13, S506A-TMPRSS13, and N400Q/N440Q-TMPRSS13 (Fig. S2*B*). Observations using either tagged or nontagged TMPRSS13 were reproducible and comparable. To investigate whether endogenously expressed TMPRSS13 is also N-linked glycosylated, we employed two different methods to assess its glycosylation in human cancer cells: (1) tunicamycin-mediated inhibition of N-linked glycosylation in live cells (58, 59), and (2) assessment of glycosylation status by PNGase F treatment of whole cancer cell lysates. For tunicamycin treatment, four cell lines were used: triple-negative breast cancer lines BT-20, HCC1937, and MDA-MB-468, and DLD1 colorectal cancer cells. Upon treatment of live cancer cells with tunicamycin for 48 h, whole cell lysates were analyzed by reducing SDS-PAGE and western blotting using the α-intra-TMPRSS13 antibody. We have previously shown that this antibody can detect endogenous TMPRSS13 in cancer cells, using siRNA-mediated TMPRSS13 silencing to confirm specificity (25, 26, 27). In untreated cells, glycosylated full-length TMPRSS13 was observed, in both the phosphorylated and nonphosphorylated forms, whereas only one band at ∼60 kDa was detected in tunicamycin-treated cells indicating that tunicamycin blocked N-linked glycosylation and phosphorylation of endogenous TMPRSS13 (Fig. S3). In parallel experiments, PNGase F was used to remove N-linked glycans from endogenous TMPRSS13 in whole cell lysates from DLD-1 and BT-20 cancer cells (Fig. S4, *A* and *B*). A similar shift in full-length TMPRSS13 migration was observed indicating the removal of endogenous N-linked glycans from the protease. Together these observations suggest that endogenous TMPRSS13 is glycosylated and that the glycosylation phenotype observed in the HEK293T cell exogenous expression model reflects a biologically relevant posttranslational modification.

### The ability of TMPRSS13 to cleave and activate its protein substrate prostasin is regulated by N-linked glycosylation

Due to the impact of N-linked glycosylation on the autoactivation of TMPRSS13, the next step was to determine if N-linked glycosylation affects the ability of TMPRSS13 to proteolytically cleave a different protein substrate. We have previously shown that endogenous TMPRSS13 levels affect endogenous prostasin protein levels in breast cancer cells and that recombinant TMPRSS13 cleaves and activates the pro-form of prostasin in HEK293T cells (26). Prostasin is a glycophosphatidylinositol (GPI)-anchored serine protease that, unlike TMPRSS13, is incapable of autoactivation, and the zymogen form requires cleavage at Arg44 by another protease in order to become proteolytically active (60). This requirement can be exploited to assess the proteolytic capability of TMPRSS13 mutants to cleave and activate prostasin zymogen.

**Figure 2:**
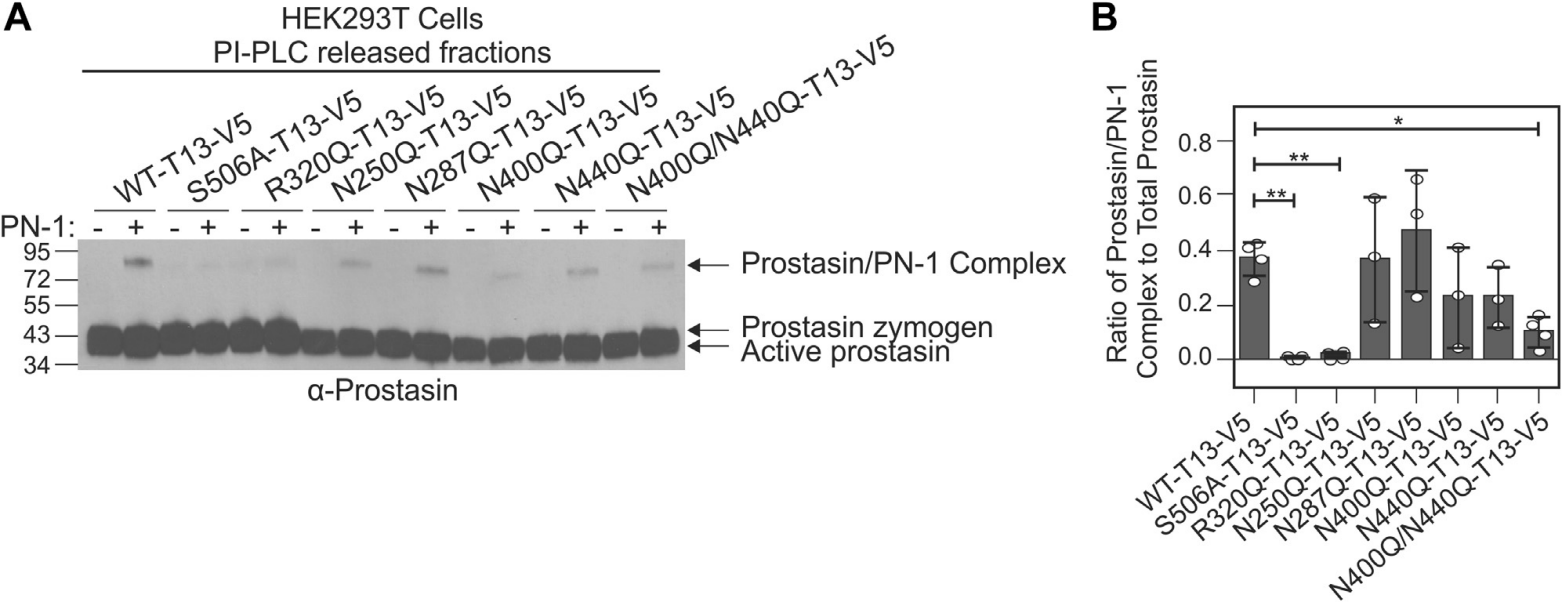
**Lack of N-linked glycosylation impairs TMPRSS13-mediated activation of prostasin.***A*, HEK293T cells were cotransfected with plasmids encoding TMPRSS13 variants and human full-length prostasin. Phosphatidylinositol-specific phospholipase C (PI-PLC) was added to cleave the glycophosphatidylinositol anchor from prostasin and release it into the supernatant. Protease nexin-1 (PN-1) was added (indicated by “+”) to form SDS-stable complexes with active prostasin. Proteins were separated by SDS-PAGE under reducing conditions using a 4 to 15% gel and proteins were detected on western blots using an anti-prostasin antibody. The prostasin zymogen and active forms, as well as the active prostasin/PN-1 complex are indicated with *arrows*. *B*, bar graph showing the ratio of the prostasin/PN-1 complex to total prostasin levels. Error bars are representative of standard deviation. One-way ANOVA with Dunnett’s multiple comparisons post-hoc test was used to determine significance of prostasin activation compared with WT-TMPRSS13. Results from at least four biological replicates are shown. ∗*p* ≤ 0.05, ∗∗ *p* ≤ 0.01.

HEK293T cells were transfected with WT-TMPRSS13-V5 and mutant expression plasmids plus full-length human prostasin, and cells were treated with phosphatidylinositol-specific phospholipase C (PI-PLC) to release prostasin into the supernatant. Subsequently, protease nexin (PN)-1, an endogenous inhibitor of prostasin that forms an SDS-stable complex with active prostasin (61), was added to the prostasin-containing supernatants. In western blot analysis, the detection of a band at ∼95 kDa corresponding to the active prostasin/PN-1 complex reflects that prostasin has been cleaved and activated by TMPRSS13 (Fig. 2*A*). In this assay, WT-TMPRSS13 is used as a positive control for prostasin activation, while S506A and R320Q mutants are used as negative controls. N250Q and N287Q mutations did not appear to affect the catalytic ability of TMPRSS13 to activate prostasin zymogen. N400Q and N440Q mutations decreased the ability of TMPRSS13 to activate prostasin, while the double mutant N400Q/N440Q showed the most significant decrease in TMPRSS13-mediated prostasin activation among the glycosylation mutants (Fig. 2*B*). These results, coupled with those from Figure 1, indicate that glycosylation in the SP domain of TMPRSS13 is critical for efficient catalytic activity for both autoactivation and cleavage/activation of a different protein substrate. Comparable results with untagged TMPRSS13 forms were observed indicating that the V5 tag does not have a discernable effect on TMPRSS13 proteolytic function (Fig. S2*C*).

### Glycosylation-deficient TMPRSS13 displays impaired localization to the cell surface and is retained in the endoplasmic reticulum

We have previously shown that WT-TMPRSS13 does not localize to the surface of HEK293T cells unless cotransfected with one of its cognate inhibitors, the Kunitz domain-containing hepatocyte growth factor activator inhibitor (HAI)-1 or HAI-2 (27). The S506A and R320Q mutants, however, readily localize to the cell surface without concomitant expression of HAIs (27). When TMPRSS13 is expressed with either HAI-1 or HAI-2, efficient cell-surface localization occurs suggesting that the cognate inhibitors facilitate TMPRSS13 localization while preventing intracellular activation (27).

**Figure 3:**
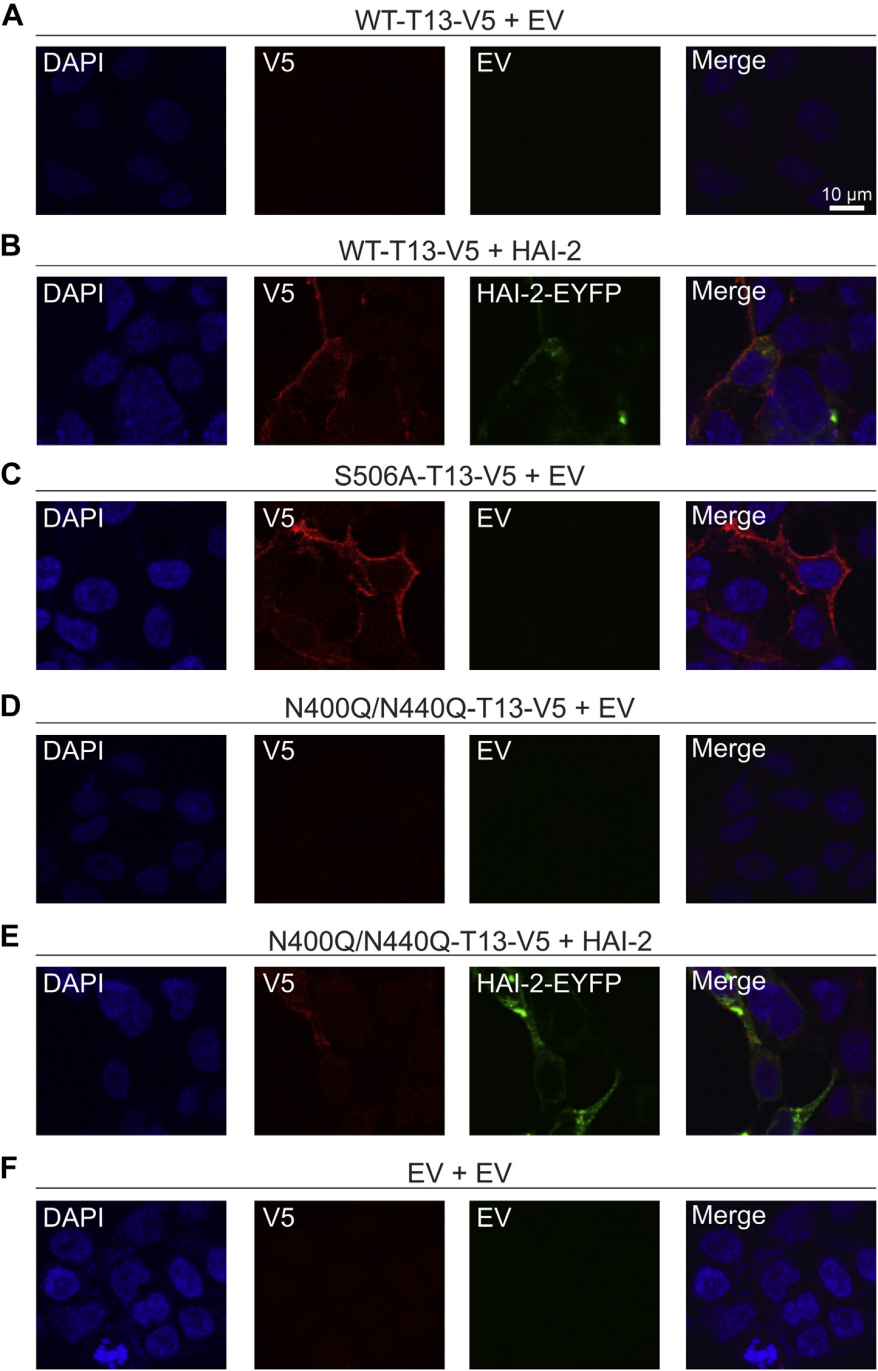
**Lack of catalytic domain glycosylation impairs TMPRSS13 cell surface localization.** Twenty-four hours after seeding onto glass coverslips, HEK293T cells were transfected for 48 h with (*A*) WT-TMPRSS13 (T13)-V5 plus empty vector (EV), (*B*) WT-TMPRSS13-V5 plus HAI-2-EYFP, (*C*) S506A-TMPRSS13-V5 plus EV, (*D*) N400Q/N440Q-TMPRSS13-V5 plus EV, (*E*) N400Q/N440Q-TMPRSS13-V5 plus HAI-2-EYFP, or (*F*) EV plus EV. Cells were fixed (no permeabilization), incubated overnight with anti-V5 antibody, and analyzed by confocal microscopy. Nuclei (DAPI) (*blue*, *A*–*F*), TMPRSS13-V5 (*red*, *A*–*F*), HAI-2 (*green*, *B* and *E*). Merged images are shown in panels on the *right*.

**Figure 4:**
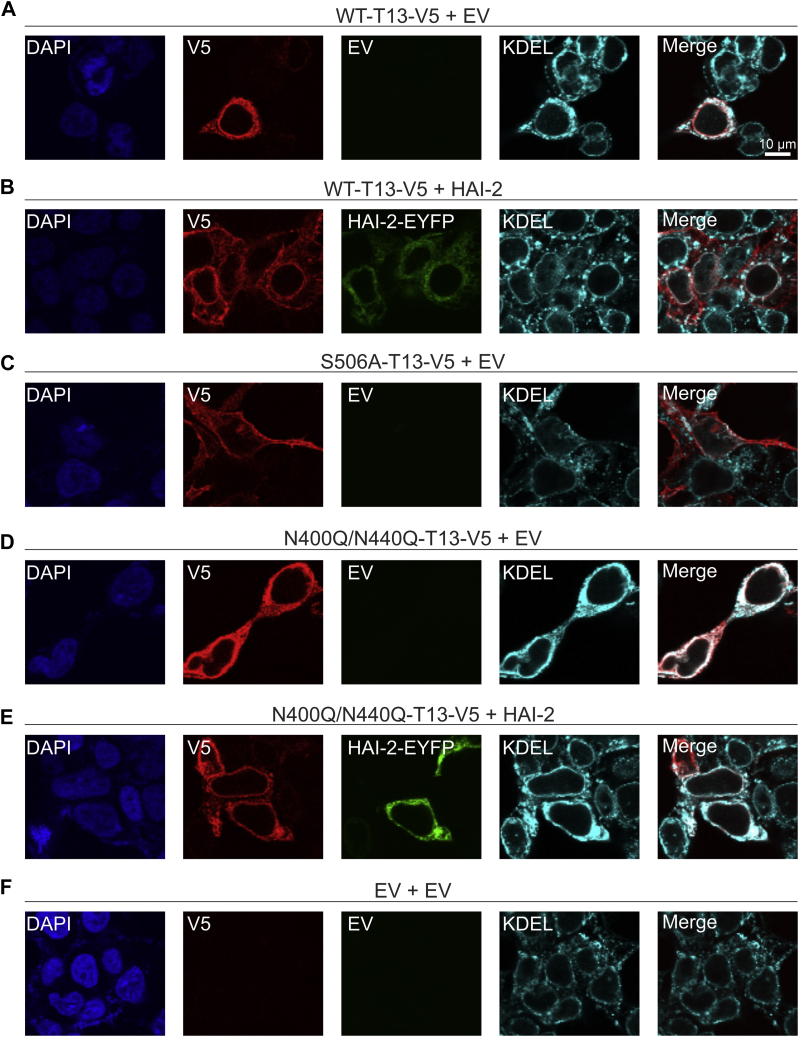
**Glycosylation deficiency causes ER retention of TMPRSS13.** Twenty-four hours after seeding onto glass coverslips, HEK293T cells were transfected for 48 h with (*A*) WT-TMPRSS13 (T13)-V5 plus empty vector (EV), (*B*) WT-TMPRSS13-V5 plus HAI-2-EYFP, (*C*) S506A-TMPRSS13-V5 plus EV, (*D*) N400Q/N440Q-TMPRSS13-V5 plus EV, (*E*) N400Q/N440Q-TMPRSS13-V5 plus HAI-2-EYFP, or (*F*) EV plus EV. Cells were fixed, permeabilized, and incubated overnight with anti-V5 to detect TMPRSS13 and anti-KDEL to detect endogenous KDEL. Nuclei (DAPI) (*blue*, *A*–*F*), TMPRSS13-V5 (*red*, *A*–*F*), HAI-2 (*green*, *B* and *E*), KDEL (*cyan*, *A*–*F*). Merged images of TMPRSS13/KDEL are shown in panels on the *right*.

To assess the role of glycosylation for TMPRSS13 cellular trafficking, immunofluorescence cell staining was performed. HEK293T cells were transfected with WT-TMPRSS13, S506A-TMPRSS13, or N400Q/N440Q-TMPRSS13, in the presence or absence of HAI-2. Cells were fixed and left nonpermeabilized to visualize cell surface staining (Fig. 3; uncropped images in Fig. S5) or were permeabilized to visualize intracellular TMPRSS13 as well as endogenous KDEL, an ER marker (Fig. 4; uncropped images in Fig. S6). As expected, in nonpermeabilized cells WT-TMPRSS13 was not detected on the cell surface when expressed alone (Fig. 3*A*). When WT-TMPRSS13 was coexpressed with HAI-2, cell surface TMPRSS13 was observed (Fig. 3*B*). S506A-TMPRSS13 localizes readily to the cell surface without requiring cotransfection with HAI-2 (Fig. 3*C*). In contrast, N400Q/N440Q-TMPRSS13 was not detected at the cell surface either in the absence or presence of HAI-2, indicating impaired cellular trafficking in this glycosylation-deficient form of TMPRSS13. To further examine the cellular fate of N400Q/N440Q, staining of permeabilized cells was performed. N400Q/N440Q was detected intracellularly and colocalized with KDEL in the absence (Fig. 4*D*) or presence (Fig. 4*E*) of HAI-2 suggesting that lack of glycosylation in the SP domain of TMPRSS13 limits the ability of TMPRSS13 to exit the ER and localize to the cell surface. The glycosylated WT-TMPRSS13, when unopposed by simultaneous transfection with HAI-2, was also retained in the ER (Fig. 4*A*) while cotransfection with HAI-2 increased cell surface localization (Fig. 4*B*).

Catalytically inactive, glycosylated S506A-TMPRSS13 efficiently localized to the surface without HAI-2 (Fig. 4*C*), suggesting that the activity status of glycosylated TMPRSS13 is an important determinant for cell surface localization. If proteolytic activity status is the only determinant for proper trafficking, it would be expected that the activity-impaired glycosylation-deficient N400Q/N440Q-TMPRSS13 would be capable of reaching the cell surface. Based on the observation that N400Q/N440Q-TMPRSS13 displays cell surface trafficking deficiency, which unlike WT-TMPRSS13 is not rescued by concomitant expression of HAI-2, we propose that glycosylation is critical for proper TMPRSS13 trafficking independent of its proteolytic activity and presence of cognate inhibitors. We considered the possibility that impaired binding of HAI-2 to N400Q/N440Q-TMPRSS13 might contribute to the lack of a rescuing effect of HAI-2. However, immunoprecipitation experiments did not reveal a significant difference between HAI-2 levels coprecipitated with WT-TMPRSS13-V5 or N400Q/N440Q-TMPRSS13-V5 (Fig. S7, *A* and *B*). It is possible though that the lack of N-linked glycans in the SP domain of TMPRSS13 changes interactions with ER chaperone proteins causing accumulation of N400Q/N440Q-TMPRSS13-V5 in the ER.

**Figure 5:**
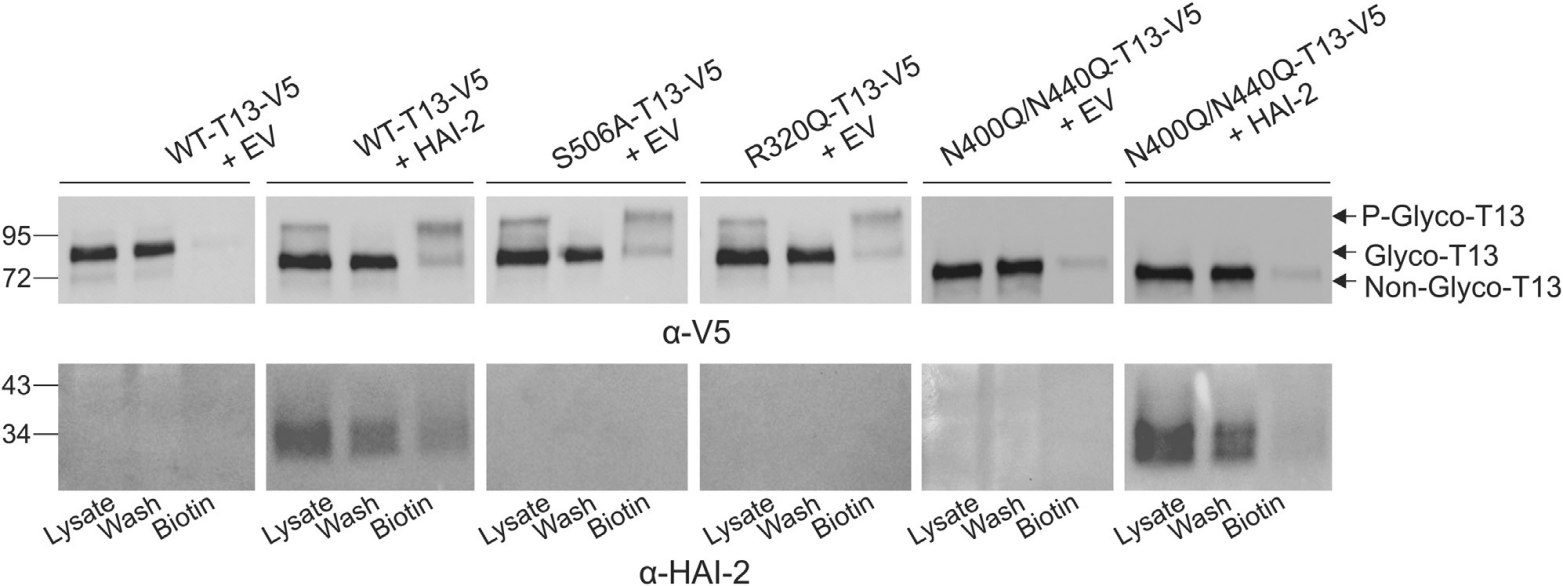
**Impairment of phosphorylation and cell surface localization in glycosylation deficient TMPRSS13.** HEK293T cells were transfected with WT-TMPRSS13 (T13)-V5, S506A-TMPRSS13-V5, R320Q-TMPRSS13 (T13)-V5, and N400Q/N440Q-TMPRSS13-V5 plasmids plus empty vector (EV) or in combination with HAI-2. Cell-surface proteins were biotin-labeled at room temperature for 30 min. Biotin-labeled proteins were precipitated with streptavidin-agarose beads for 1 h at 4 °C. Beads were pelleted and supernatants containing nonbiotinylated proteins were collected (*wash*). Beads were washed five times in PBS, then biotin-labeled proteins were eluted from beads by treatment with Laemmli buffer with 5% 2-mercaptoethanol. Whole cell lysate input (*lysate*), nonbiotinylated proteins (*wash*), and biotin-labeled proteins (*biotin*) were separated by SDS-PAGE under reducing conditions using 10% gels. Proteins were detected by western blotting using anti-V5 (TMPRSS13) and anti-HAI-2 antibodies. Glyco-T13, glycosylated TMPRSS13; Non-glyco-T13, nonglycosylated TMPRSS13; P-glyco-T13, phosphorylated and glycosylated TMPRSS13.

To further validate the immunocytochemistry findings using a biochemical approach, we analyzed lysates from cells expressing WT or mutant TMPRSS13 in combination with cell-surface biotinylation analysis using a membrane-impermeable biotinylation reagent. HEK293T cells were transfected with WT-TMPRSS13 or mutant plasmids, in the presence or absence of HAI-2. Cell-surface proteins were biotin-labeled and pulled down using streptavidin-agarose beads and samples were subsequently analyzed by SDS-PAGE and western blotting (Fig. 5). As expected, there is a predominance of the HMW (phosphorylated) form of TMPRSS13 at the cell surface, as previously seen with WT-TMPRSS13 coexpressed with HAI-2 and with the S506A and R320Q mutants cotransfected with empty vector (EV) (27). In addition, the “wash” fractions containing nonbiotinylated proteins show minimal or no phosphorylated TMPRSS13, indicating that the majority of phosphorylated TMPRSS13 is localized to the cell surface. WT-TMPRSS13 shows minimal cell surface localization when unopposed by HAI-2 while cell-surface TMPRSS13 is readily detected upon coexpression with HAI-2. No discernable differences between WT-TMPRSS13 (with or without HAI-2) and SRCR domain glycosylation site mutants (N250Q and N287Q) were observed (data not shown). Importantly, for N400Q/N440Q-TMPRSS13 a cell-surface phosphorylated form is undetectable in both the presence and absence of HAI-2. These data, in combination with the immunofluorescent cell staining, indicate that ER retention of glycosylation-deficient TMPRSS13 causes impairment of trafficking to the cell surface, independent of inhibitor expression.

### Endogenous TMPRSS13 is modified by N-linked glycosylation and phosphorylation

**Figure 6:**
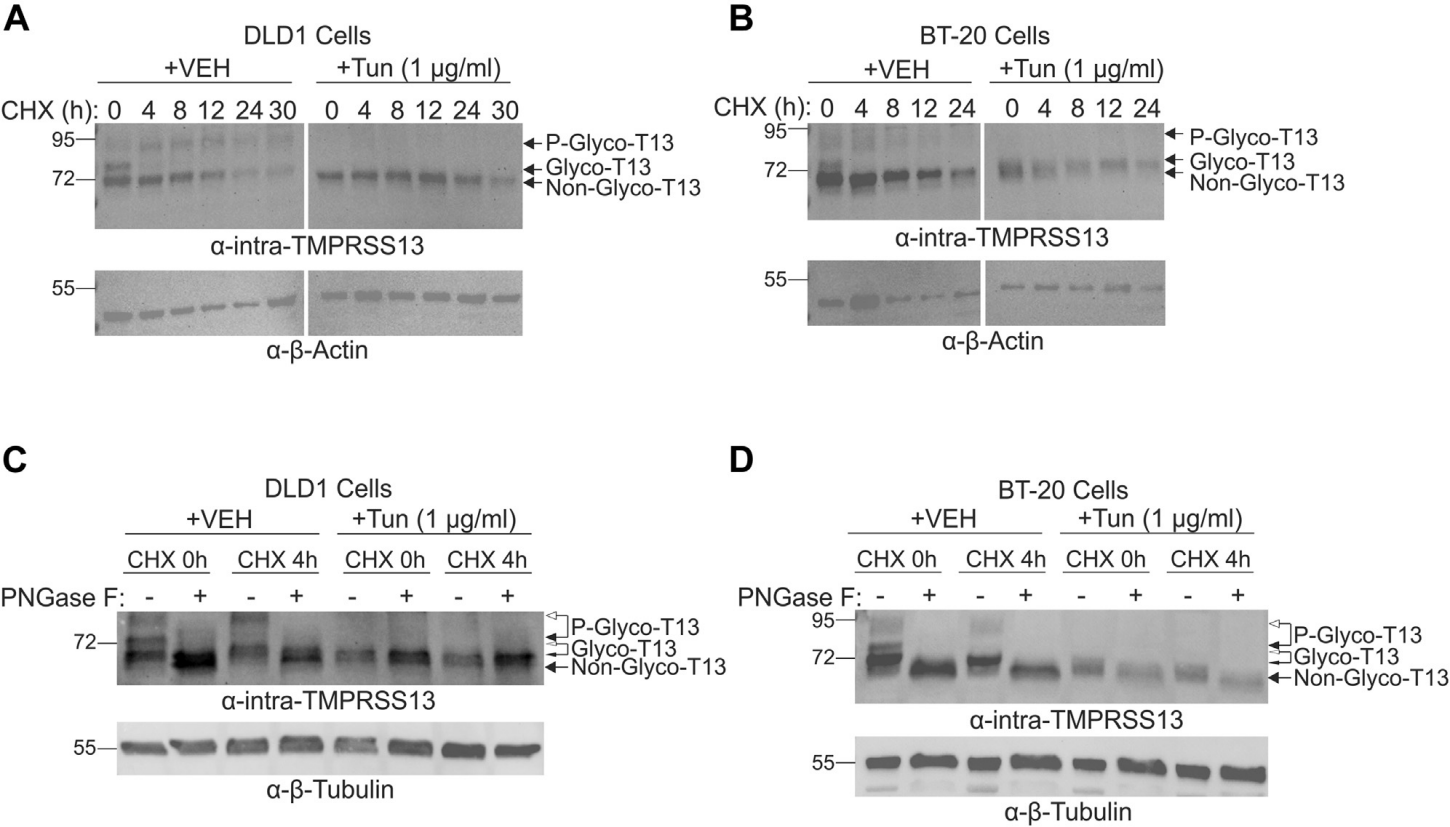
**N-linked glycosylation determines cellular stability and phosphorylation status of endogenous TMPRSS13.***A*, DLD1 colorectal cancer cells or *B*, BT-20 breast cancer cells were treated with 1 μg/ml Tunicamycin (Tun) or vehicle control (VEH; DMSO) for 24 h, then cycloheximide (CHX) was added for (*A*) 0 to 30 or (*B*) 0 to 24 h. Proteins in whole cell lysates were separated by SDS-PAGE under reducing conditions using 10% gels and detected on western blots using anti-intra-TMPRSS13 and anti-β-actin antibodies. Whole cell lysates from (*C*) DLD1 or (*D*) BT-20 cells treated with tunicamycin or vehicle control and cycloheximide for 0 to 4 h were subsequently treated with PNGase F prior to SDS-PAGE under reducing conditions (indicated by “+”) using 10% gels. Lanes with lysates without PNGase F treatment are indicated by “−”. Proteins were detected by western blotting using anti-intra-TMPRSS13 and anti-β-tubulin antibodies. The *white arrowheads* connected to *black arrowheads* indicate the mobility shift upon PNGase F treatment. Glyco-T13, glycosylated TMPRSS13; Non-glyco-T13, non-glycosylated TMPRSS13; P-glyco-T13, phosphorylated and glycosylated TMPRSS13.

To investigate the role of glycosylation for endogenous TMPRSS13 turnover and phosphorylation status, BT-20 and DLD1 cells were treated with cycloheximide and/or tunicamycin followed by western blot analysis of whole-cell lysates. HAI-1 and HAI-2 are endogenously expressed in both cell lines (27). Importantly, both cell lines express a full-length, glycosylated TMPRSS13 form (denoted as Glyco-T13 in Fig. 6) and a lower-molecular-weight TMPRSS13 form (non-Glyco-T13) that are detected prior to treatment with cycloheximide (Fig. 6, *A*–*D* left panels, vehicle, 0 h cycloheximide; Fig. S4, *A* and *B*) or tunicamycin (Fig. 6, *A*–*D* right panels, vehicle, 0 h cycloheximide; Fig. S3). We infer that this lower-molecular-weight variant represents a hypo- or non-glycosylated form of TMPRSS13, which is supported by the findings that only this form is detected upon PNGase F treatment (Fig. 6, *C* and *D*) or tunicamycin treatment (Fig. 6, *A*–*D* right panels, tunicamycin). The HMW phosphorylated form of TMPRSS13 (P-Glyco-T13) appears to be glycosylated as demonstrated by its absence (nondetectable) in tunicamycin-treated cells and a shift in mobility upon PNGase F treatment (Fig. 6, *C* and *D*). Detection of endogenous full-length glycosylated TMPRSS13 (Glyco-T13) rapidly decreases within the first 4 h of cycloheximide treatment (Fig. 6, *A*–*D*), while nonglycosylated TMPRSS13 (non-Glyco-T13) is detectable for up to 30 h (Fig. 6, *A* and *B*). The persistent detection of nonglycosylated TMPRSS13 may reflect increased ER retention, as observed with the N400Q/N440Q-TMPRSS13 mutant in Figure 4. Interestingly, the HMW phosphorylated and glycosylated form of TMPRSS13 (P-Glyco-T13) also appears to have increased stability compared with the nonphosphorylated glycosylated form and can be detected for up to 30 h in DLD1 cells and 12 h in BT20 cells (Fig. 6, *A* and *B*). Additionally, tunicamycin treatment eliminates detection of the phosphorylated form of TMPRSS13 (Fig. 6, *A*–*D* and Fig. S3). The fast turnover of Glyco-T13 may reflect conversion to P-Glyco-T13 with subsequent transport to the secretory pathway and cell surface. Cycloheximide likely abrogates generation of new Glyco-T13 as it has been shown that N-glycosylation is inhibited by cycloheximide within approximately 30 min due to a lack of newly synthesized acceptor polypeptides (62).

Together the results indicate that proper glycosylation of endogenous TMPRSS13 is necessary for its phosphorylation and possibly localization to the cell surface.

## Discussion

In this study we determined that the N-linked glycosylation of TMPRSS13 in its catalytic SP domain plays an important role in its cell surface localization as well as its function as an enzyme. The process of N-linked glycosylation is highly conserved and occurs on the majority of eukaryotic proteins that are translated across the rough ER (39, 63). The functional roles of N-linked glycans are plentiful, including contributions to proper protein folding, protein quality control, intracellular trafficking, and protein stability (63, 64). Proteins that are unfolded or misfolded due to a lack of glycosylation are often recognized by ER chaperones and degraded by the ER-associated degradation system (ERAD) (65).

Several TTSP family members are known to harbor N-linked glycosylation sites that are important for their zymogen activation and cellular localization (44, 46, 49, 50). Mutation of N-linked glycosylation sites in the catalytic domain of TMPRSS13 significantly reduces its proteolytic activity, demonstrated by a lack of autoactivation and a decrease in TMPRSS13-mediated prostasin activation cleavage. Furthermore, we show that glycosylation in the SP domain is essential for phosphorylation and cell surface localization. In contrast, mutation of glycosylation sites in the SRCR domain did not have any significant effects on TMPRSS13 activity and trafficking. This reliance on N-linked glycans for adequate zymogen activation/catalytic activity and cell trafficking has been shown in other TTSPs including corin, hepsin, matriptase, and matriptase-2 (44, 47, 49, 50). Human corin harbors 19 N-glycosylation sites in its extracellular region (66). Corin does not autoactivate; however, N-glycosylation at Asn1022 (N1022), the only N-glycosylation site in the protease domain of human corin, is critical for zymogen activation by proprotein convertase subtilisin/kexin-6 (PCSK6) (50, 51, 67, 68, 69). Abolishing N-glycosylation at N1022 also reduces the cell surface expression of corin (50). Matriptase-2 has seven N-glycosylation sites, but unlike TMPRSS13 and corin, none of these are located in the SP domain (49). Matriptase-2 is capable of autoactivation and mutation of N216Q, N453Q, and N518Q, but not the other mutants, caused impaired zymogen activation. All three of these N-glycosylation sites are located away from the activation cleavage site. Mutations at two separate N-glycosylation sites in the closely related TTSP, matriptase, one in the CUB domain and another in the SP domain, also impaired matriptase activation (47, 48). It was proposed that N-glycans, at least in some cases, do not directly affect substrate binding but may be important for maintenance of a proper protein conformation required for the autoactivation of these TTSPs (48). To this end, the TTSP hepsin, like matriptase-2, has no glycosylation sites in the SP-domain and harbors a single N-glycosylation site at Asn112 in the SRCR domain (44), yet mutation of the SRCR domain site leads to impaired hepsin zymogen activation, intracellular trafficking, and cell surface expression (44).

The requirement of N-glycosylation of a particular protease may depend on its expression level and specific cell environments including presence of cognate inhibitors and/or ER chaperones. Glycosylation in the SP domain of TMPRSS13 is critical for autoactivation; however, the precise mechanism is still unknown. For matriptase, it has been proposed to be a transactivation process initiated by interaction between zymogen forms, which leads to activation of the protease and that HAI-1 and HAI-2 regulate this trans(auto)-activation (48, 70). In HEK293T cells where endogenous TMPRSS13, HAI-1, or HAI-2 proteins ((27) and data in this study) are not detected, autoactivation of recombinant TMPRSS13 and activation of the protein substrate, prostasin, are observed without concomitant expression of HAI-1/HAI-2. Importantly, HAIs are essential for WT-TMPRSS13 trafficking to the cell surface ((27) and data in this study). The impaired N400Q/N440Q-TMPRSS13 transport to the cell surface accompanied by ER/Golgi accumulation was not rescued by coexpression with HAI-2, as shown by both immunocytochemistry and cell surface biotinylation assays. However, no significant impairment of the mutant’s ability to bind HAI-2 was detected. It can be speculated that one or more additional binding partners are involved in proper TMPRSS13 trafficking and that binding to these partners is affected by lack of glycans in the SP domain. An example of this was observed in studies of corin where the SP domain N1022Q mutant displayed impaired cell surface localization (46). Increased binding of the N1022Q mutant to calnexin, a chaperone that specifically acts to retain unfolded or unassembled N-linked glycoproteins in the ER, was observed, and it was proposed that N-glycosylation in the protease domain mediates calnexin-assisted protein folding with subsequent ER exiting and transport to the cell surface (46). Studies are underway to identify TMPRSS13-binding partners critical for cellular trafficking.

Importantly, our observations from exogenously expressed TMPRSS13 in HEK293T cells extend to endogenous forms of TMPRSS13 in cancer cell lines. We detected glycosylated forms of endogenous TMPRSS13 in human cancer cells using tunicamycin-mediated inhibition of the N-glycosylation process and PNGase F-mediated removal of N-glycans. These studies revealed the presence of three major forms in total cell lysates, which represent non-glycosylated, glycosylated, and glycosylated/phosphorylated TMPRSS13. The observation that the phosphorylated form of TMPRSS13 is not detected upon tunicamycin treatment suggests that N-glycosylation is critical for phosphorylation. The detection of different endogenous glycosylation variants of TMPRSS13 with differential stability and phosphorylation status in breast and colorectal cancer cells is interesting because proteomic/glycomic studies have demonstrated that some cancer-associated proteases exhibit altered glycosylation patterns with functional implications in malignancies. These studies include the secreted kallikrein-related peptidases (KLKs), a family of serine proteases (71, 72). For example, changes in KLK3 glycosylation patterns were observed in samples from patients with prostate cancer compared with benign prostatic hyperplasia (73). Furthermore, glycosylation of KLK2 has a significant effect on protease activity against small synthetic substrates (74). Studies of matriptase proposed that the pro-metastatic effect of the cancer-associated glycosyltransferase N-acetylglucosaminyltransferase V (GnT-V) in gastric cancer cells was mediated by modification and stabilization of active matriptase upon addition of β1 to 6 GlcNAc branching (75). In a follow-up study, analysis of matriptase and GnT-V in thyroid cancer tissues suggested that posttranslational modification by GnT-V contributes to regulation of matriptase levels (76). It is plausible that glycosylation of TMPRSS13 is critical for its demonstrated pro-oncogenic properties (25, 26) by modulation of activity, stability, and cell-surface localization and therefore warrants further studies.

TMPRSS13 is unique among the TTSP family members by harboring a long cytoplasmic tail that is highly disordered and phosphorylated (27). The intracellular domain of TMPRSS13 contains a total of 30 serine residues, 12 threonine residues, and one tyrosine residue (27) of which at least 14 are phosphorylated (Martin and List, unpublished data). On the cell surface, the phosphorylated form is the most prevalent, and coexpression of HAI-1 or HAI-2 with WT-TMPRSS13 promotes TMPRSS13 phosphorylation and cell-surface localization (27). It is not yet known whether phosphorylation is required for cell-surface localization or whether phosphorylation exclusively takes place on cell-surface localized TMPRSS13. Therefore, the lack of phosphorylated TMPRSS13 upon tunicamycin treatment may reflect an indirect effect by impairing transport of TMPRSS13 to the cellular compartment where phosphorylation takes place, possibly at the cell surface. Though posttranslational modification of TMPRSS13 by N-glycosylation occurs in its extracellular region while phosphorylation occurs in its intracellular region, a direct effect of glycosylation on phosphorylation cannot entirely be ruled out. The role that phosphorylation plays in the activity and trafficking of TMPRSS13 is currently under investigation.

Recently, it has been shown that TMPRSS13 has antiapoptotic properties (25, 26) and that the protease promotes cancer progression *in vivo* (26). Furthermore, TTSPs including TMPRSS13 have garnered significant attention in virology because they can enhance viral entry driven by the spike proteins of the highly virulent MERS-CoV, SARS-CoV, and SARS-CoV-2 (38). The active form of TMPRSS13 is expressed in human lung as well as nasal tissue, and siRNA-mediated knockdown of TMPRSS13 in Calu-3 human lung cancer cells resulted in a significant reduction in SARS-CoV-2 replication (38). Therefore, TMPRSS13 with its accessibility on the cell surface represents a candidate target for the development of inhibitors for treating cancer and viral infections.

In summary, this study identifies that the N-linked glycosylation status of the SP domain of TMPRSS13 is critical for its zymogen autoactivation, proteolytic activity toward the protein substrate prostasin, phosphorylation, and cellular localization. These new insights lay the groundwork for future functional and mechanistic studies defining the role of posttranslational modifications of TMPRSS13 under various pathophysiological conditions.

## Experimental procedures

### Cell lines and culture conditions

HEK293T, COS7, and MDA-MB-468 cells (all from ATCC) were cultured in Dulbecco’s modified eagle media (Gibco/Thermo Fisher Scientific) supplemented with 10% fetal bovine serum (FBS) (Atlanta Biologicals), 10 units/ml Penicillin, and 10 μg/ml streptomycin (Gibco, Life Technologies). HCC1937 cells (ATCC) were cultured in RPMI + L-GLUT media (RPMI-1640 media with 2 mM L-glutamine) supplemented with 1.5 g/l sodium bicarbonate, 4.5 g/l glucose, 10 mM HEPES, 1 mM sodium pyruvate, 10% FBS, and 10 units/ml Penicillin and 10 μg/ml streptomycin. BT-20 cells (ATCC) were cultured in Eagle’s + NEAA media (Eagle’s minimal essential medium with 2 mM L-glutamine and Earle’s balanced salt solution adjusted to contain 1.5 g/l sodium bicarbonate, 0.1 mM nonessential amino acids, 1 mM sodium pyruvate, and 10% FBS). DLD1 cells (ATCC) were grown in RPMI 1640 media + L-GLUT media adjusted to contain 10% FBS.

### Western blotting

Cultured human cells were washed three times with ice-cold PBS and lysed in-well using ice-cold RIPA buffer (150 mM NaCl; 50 mM Tris/HCl, pH 7.4, 0.1% SDS; 1% NP-40) with protease inhibitor cocktail (Sigma Aldrich) and phosphatase inhibitor cocktail (Sigma Aldrich), and cleared by centrifugation at 12,000*g* at 4 °C. Protein concentrations were determined using a Pierce BCA Protein Assay Kit (Thermo Fisher Scientific). Proteins were separated by SDS-PAGE under reducing conditions using 10% or 4 to 15% Mini-Protean gels or Criterion TGX midi gels (Bio-Rad) and blotted onto PVDF membranes. Membranes were blocked with 5% (w/v) dry milk powder in TBS-T (Tris-buffered saline, 0.1% Tween-20) for 1 h at room temperature and subsequently incubated overnight at 4 °C in primary antibodies diluted in 5% dry milk powder/TBS-T. Primary antibodies used for western blotting included rabbit anti-TMPRSS13 raised against a recombinant protein fragment corresponding to a region within amino acids 195 and 562 of human TMPRSS13 (anti-extra-TMPRSS13) (PA5-30935, Thermo Fisher Scientific and Life Technologies, Inc); rabbit anti-TMPRSS13 raised against an epitope within the first 60 amino acids of human TMPRSS13 (anti-intra-TMPRSS13) (ab59862, Abcam); mouse-anti-V5 (R960-25, Thermo Fisher Scientific and Life Technologies, Inc); goat anti-HAI-2 (AF1106, R&D Systems Inc); mouse anti-prostasin (612173, BD Biosciences), rabbit anti-Histone H3 (D1H2, Cell Signaling Technology), mouse anti-beta-tubulin (E7-c, Developmental Studies Hybridoma Bank), and mouse anti-beta-actin (NB600-501, Novus Biologicals). Secondary antibodies included goat anti-rabbit (12-348, Millipore), goat anti-mouse (AP181P, Millipore), and rabbit anti-goat (31403, Thermo Fisher Scientific) HRP-conjugated antibodies. Detection of antibodies was performed using ECL Western Blotting substrate or Super-Signal West Femto Chemiluminescent Substrate (Pierce, Thermo Fisher Scientific). After detection, PVDF membranes were stripped using Restore Western Blot Stripping Buffer (Thermo Fisher Scientific) for 15 min at room temperature prior to reprobing with a different primary antibody.

### Cloning of full-length TMPRSS13 plasmid constructs

WT-TMPRSS13-V5, S506A-TMPRSS13-V5, R320Q-TMPRSS13-V5, and untagged WT-TMPRSS13 and S506A-TMPRSS13 constructs were generated as previously described (27). Point mutations using WT-TMPRSS13-V5 as a template for N250Q-TMPRSS13-V5, N287Q-TMPRSS13-V5, N400Q-TMPRSS13-V5, and N440Q-TMPRSS13-V5 were generated using the Q5 Site-Directed Mutagenesis Kit (New England Biolabs). The N400Q/N440Q-TMPRSS13-V5 mutant plasmid was synthesized by the GenScript company. Primers used for N250Q mutagenesis were 5′-CAGCAACTGGCAAGACTCCTACTC-3′ and 5′-CTACAGATGGGAAGCCAC-3′. Primers used for N287Q mutagenesis were 5′-CTTGAGATACCAATCCACCATCCAG-3′ and 5′- ATTGAGAAGCTG TTGGCAAAATC-3′. Primers used for N400Q mutagenesis were 5′-CATCAACAGCCAATACACCGATGAG-3′ and 5′-ATGATCTCGGCAATGGAG-3′. Primers used for N440Q mutagenesis were 5′-CTTTAGCCTCCAAGAGACCTGCTGG-3′ and 5′-GTCTGTCCATGCATGGGG-3′. For untagged N400Q/N440Q-TMPRSS13 construct, point mutations were made using WT-TMPRSS13 as a template and primers for N400Q and N440Q mutagenesis. Transformation of all vectors was performed in NEB 5-alpha Competent *E. coli* cells (New England Biolabs), and positive clones were isolated and amplified using standard techniques.

### Transient transfections with TMPRSS13 expression vectors

Transfections of HEK293T cells were performed using Lipofectamine LTX reagent with PLUS reagent according to the manufacturer’s instructions (Invitrogen, Life Technologies, Inc). Transfection was performed with 500 ng of plasmid DNA for single transfections or 1 μg of DNA total for cotransfections. Vectors included in transfections were pcDNA3.1-TMPRSS13 vectors, empty vector pcDNA3.1, and pcDNA3.1-HAI-2 (77). The HAI-2 vector was kindly provided by Dr Stine Friis, University of Copenhagen.

### Prostasin/PN-1 complex formation assay

Forward transfection of the mammalian expression vector pIRES2-EGFP containing full-length human prostasin cDNA and TMPRSS13 plasmids (WT, S506A, R320Q, N250Q, N287Q, N400Q, N440Q, N400Q/N440Q) was performed in HEK293T cells. Transfection was performed with 1 μg of total plasmid DNA for cotransfections per well using Lipofectamine LTX reagent with PLUS reagent according to the manufacturer’s instructions (Invitrogen, Life Technologies, Inc). For phosphatidylinositol-specific phospholipase C (PI-PLC) treatment, washed cells were mechanically lifted from the plates by gentle pipetting, incubated with 1 unit/ml PI-PLC (Sigma-Aldrich) in PBS for 4 h at 4 °C, then centrifuged for 10 min at 1000*g*, and the supernatant containing the PI-PLC-released proteins was collected. For complex formation, PN-1 derived from murine sperm (described in (61)) was added for 1 h at 37 °C in 50 mM Tris, 100 nM NaCl pH 8.5. Proteins were analyzed by reducing SDS-PAGE and western blot analysis.

### Tunicamycin and cycloheximide treatment

Tunicamycin (T7765, Sigma Aldrich) was reconstituted in DMSO to a concentration of 10 mg/ml. Cancer cell lines were plated and allowed to adhere to plates and reach ∼80% confluence. Cells were subsequently treated with tunicamycin at a final concentration of 1 μg/ml for 48 h. Vehicle-treated cells received an equivalent volume of DMSO. When tunicamycin treatment was performed with cycloheximide treatment, DLD1 and BT-20 cells were treated for 24 h with tunicamycin prior to treatment with 75 μg/ml of cycloheximide (C-1189, AG Scientific) for up to 30 h. Following treatment, cells were washed three times in PBS and lysed in-well using RIPA buffer.

### Deglycosylation of TMPRSS13

Proteins in lysates prepared as indicated above were deglycosylated using the PNGase F deglycosylation kit according to the manufacturer’s instructions (New England Biolabs).

### Biotin labeling of cell surface proteins

Forty-eight hours posttransfection, HEK293T cells expressing TMPRSS13, empty vector (EV), and/or HAI-2 constructs were washed three times with PBS. Cells were then gently detached and resuspended in 1.0 ml of PBS, and EZ-Link Sulfo-NHS-SS-Biotin (Thermo Scientific) was added for a final concentration of 800 μM. Cells were biotin-labeled for 30 min at room temperature. After biotin labeling, cells were pelleted and the biotinylation reaction was quenched by washing the cells three times with PBS containing 100 mM glycine. Cells were then lysed in RIPA buffer supplemented with protease inhibitor mixture (Sigma), and protein concentrations were quantitated. In total, 120 μg of protein was added to 40 μl of streptavidin-agarose (Sigma) in a final reaction volume of 200 μl and rotated at 4 °C for 60 min. Beads were pelleted by centrifugation at 800*g*, and supernatant containing nonbiotinylated proteins was collected (wash). Beads were washed five times with cold PBS and subsequently treated with 60 μl of Laemmli sample buffer with 5% 2-mercaptoethanol and boiled for 5 min prior to SDS-PAGE.

### Immunoprecipitation

Forty-eight hours posttransfection, HEK293T cells expressing TMPRSS13, empty vector (EV), and/or HAI-2 were lysed with RIPA lysis buffer with protease inhibitor mixture. One microliter of primary mouse anti-V5 (R960-25, Thermo Fisher Scientific) was added to 150 μg of protein lysates, and lysis buffer was added for a total reaction volume of 250 μl. Lysates were then rotated at 4 °C for 60 min. After immunoprecipitation, 30 μl of EZview Red Protein A affinity gel (Sigma) was added to the reaction per the manufacturer’s protocol. Samples were then rotated at 4 °C for 60 min, then beads were pelleted at 4 °C and washed five times with cold PBS, pH 7.5. After the final wash, 60 μl of 2× Laemmli buffer with 5% 2-mercaptoethanol was added, and samples were analyzed by SDS-PAGE and western blotting.

### Immunocytochemistry

Cell imaging was performed using HEK293T cells transfected with human full-length TMPRSS13 vectors, empty vector pcDNA3.1, or HAI-2-EYFP (enhanced yellow fluorescent protein) (78). Cells were seeded on coverslips and allowed to adhere and grow overnight. Cells were transiently transfected, and 48 h post transfection, media was removed and cells were fixed in Z-Fix (ANATECH LTD) for 15 min at room temperature. For permeabilized samples, cells were treated with 0.05% Triton X-100 in PBS for 15 min on ice. Cells were then blocked in 5% BSA in PBS for 1 h prior to addition of primary antibodies. TMPRSS13-V5 was detected using a monoclonal anti-V5 antibody (Invitrogen, Life Technologies, Inc) or an isotype control antibody. KDEL was detected using a monoclonal anti-KDEL antibody (PA1-013, Invitrogen, Life Technologies, Inc) or an isotype control antibody. After incubation with primary antibodies overnight at 4 °C, cells were washed in PBS and secondary Texas Red-conjugated goat anti-mouse and AlexaFluor-647-conjugated goat anti-rabbit antibodies (Invitrogen, Life Technologies, Inc) were used to detect TMPRSS13-V5 or KDEL. Cells were washed with PBS and mounted with ProLong Diamond Antifade Mountant with DAPI (Invitrogen). Confocal images were acquired on the Leica SP5 scope at the Microscopy Imaging and Cytometry Resources Core at Wayne State University School of Medicine. Acquired permeabilized images were pseudo-colored and merged using ImageJ image analysis (79).

### Statistical analyses

GraphPad Prism software was used for all statistical analyses. For statistical comparisons between three or more groups, one-way ANOVA was used with Dunnett’s post hoc analysis. For statistical comparisons between two groups, Student’s *t* test was used. All statistical analyses are representative of at least three biological replicates.

## Data availability

All data supporting this article are included within the main text and supporting information.

## Supporting information

This article contains supporting information.

## Conflict of interest

The authors declare that they have no conflicts of interest with the contents of this article.
