# Supplementary material for: Posttranslational modifications of serine protease TMPRSS13 regulate zymogen activation, proteolytic activity, and cell surface localization
Source: J Biol Chem. 2021 Sep 22;297(4):101227. doi: 10.1016/j.jbc.2021.101227 (PMC8503615; doi:10.1016/j.jbc.2021.101227)

**Post-translational modifications of serine protease TMPRSS13 regulate zymogen activation, proteolytic activity, and cell surface localization**

Carly E. Martin[1,2], Andrew S. Murray[1,2,3], Kimberley E. Sala-Hamrick[1], Jacob R. Mackinder[1], Evan C. Harrison[1], Joseph G. Lundgren[1,2], Fausto A. Varela[1,4], Karin List[1,2]

Department of Pharmacology[1], Department of Oncology [2], Wayne State University, Detroit, MI, USA; Division of Hematological Malignancies and Cellular Therapy[3], Duke University, Durham, NC, USA; Department of Biochemistry and Molecular Biology[4], University of Kansas Medical Center, Kansas City, KS, USA.

Keywords: serine protease, N-linked glycosylation, phosphorylation, cell surface protein, protease inhibitor, type II transmembrane serine protease, TTSP, TMPRSS13, HAI-2

Running title: Post-translational modifications regulate TMPRSS13 functions

Corresponding author:

Karin List, Ph.D.
Department of Pharmacology and Department of Oncology
Gordon H. Scott Hall of Basic Medical Sciences Room 6332
540 East Canfield, Detroit
Michigan 48201
Phone: 313-577-1034
E-mail: klist@med.wayne.edu

**Supporting Information**

**Figure S1.** HEK293T cells were transfected with TMPRSS13 constructs and proteins from cell lysates were separated by SDS-PAGE under reducing conditions using a 4-15% gel. Lanes with protein extracts treated with PNGase F prior to SDS-PAGE are indicated by "+" and those that received no treatment are indicated by "-". Proteins were detected using anti-extra-TMPRSS13, anti-intra-TMPRSS13, and anti-histone H3 antibodies. Arrows to the right of the western blots indicate TMPRSS13 bands defined in Figure 1 and their representative schematics. The white arrowheads connected to black arrowheads indicate the mobility shift upon PNGase F treatment.

**Figure S2**. *A*, HEK293T and Cos7 cells were transfected with WT-TMPRSS13-(T13)-V5, S506A-TMPRSS13-V5 or N400Q/N440Q-TMPRSS13-V5. Proteins in whole cell lysates were separated by SDS-PAGE using 10% gels and detected on Western blots using anti-extra-TMPRSS13, anti-intra-TMPRSS13 and anti-β-actin antibodies. *B*, HEK293T cells were transfected with untagged full-length WT-TMPRSS13 (T13), S506A-TMPRSS13, N400Q/N440Q-TMPRSS13, or empty vector (EV). Proteins in whole cell lysates were separated by SDS-PAGE using 10% gels and detected by western blotting using anti-extra-TMPRSS13, anti-intra-TMPRSS13 and anti-β-actin antibodies. P-glyco-T13, phosphorylated and glycosylated TMPRSS13; Glyco-T13, glycosylated TMPRSS13; Non-glyco-T13, non-glycosylated TMPRSS13. *C*, HEK293T cells were transfected with untagged TMPRSS13 constructs and human full-length prostasin. Cells were collected in PBS, then phosphatidylinositol-specific phospholipase C (PI-PLC) was added to cleave the glycophosphatidylinositol anchor from prostasin and release it into the supernatant. Protease nexin-1 (PN-1) was added (indicated by "+") to form SDS-stable complexes with active prostasin. Supernatant proteins were separated

by SDS-PAGE under reducing conditions using a 4-15% gel and detected by western blotting using an anti-prostasin antibody. The prostasin zymogen and active forms, as well as the active prostasin/PN-1 complex are indicated with arrows.

**Figure S3**. Breast cancer (BT-20, HCC1937, MDA-MB-468) and colorectal cancer (DLD1) cell lines were treated for 48 hours with 1 µg/ml Tunicamycin (indicated with "+") or vehicle control (DMSO, indicated with "-"). Proteins in whole cell lysates were separated by SDS-PAGE under reducing conditions using a 10% gel and detected by western blotting using anti-intra-TMPRSS13 and anti-β-actin antibodies. P-glyco-T13, phosphorylated and glycosylated TMPRSS13; Glyco-T13, glycosylated TMPRSS13; Non-glyco-T13, non-glycosylated TMPRSS13.

**Figure S4**. Proteins from (*A*) DLD1 or (*B*) BT-20 cell lysates were separated by SDS-PAGE under reducing conditions using 10% gels. Lanes with protein extracts treated with PNGase F prior to SDS-PAGE are indicated by "+" and those that received no treatment are indicated by "-". Proteins were detected by western blotting using anti-intra-TMPRSS13 and anti-β-tubulin antibodies. The white arrowheads connected to black arrowheads indicate the mobility shift upon PNGase F treatment.

**Figure S5.** Uncropped images from Figure 3. 24 hours after seeding onto glass coverslips, HEK293T cells were transfected for 48 hours with (*A*) WT-TMPRSS13 (T13)-V5 plus EV, (*B*) WT-TMPRSS13-V5 plus HAI-2-EYFP, (*C*) S506A-TMPRSS13-V5 plus EV, (*D*) N400Q/N440Q-TMPRSS13-V5 plus EV, (*E*) N400Q/N440Q-TMPRSS13-V5 plus HAI-2-

EYFP, or (*F*) EV plus EV. Cells were fixed (no permeabilization), incubated overnight with anti-V5 antibody, and analyzed by confocal microscopy. Nuclei (DAPI) (*blue, A-F*), TMPRSS13-V5 (*red, A-F*), HAI-2 (*green, B and E*). Merged images are shown in panels on the right. Yellow boxes indicate the regions of the images that were cropped for Figure 3.

**Figure S6.** Uncropped images from Figure 4. 24 hours after being seeded onto glass coverslips, HEK293T cells were transfected for 48 hours with (*A*) WT-TMPRSS13 (T13)-V5 plus empty vector (EV), (*B*) WT-TMPRSS13-V5 plus HAI-2-EYFP, (*C*) S506A-TMPRSS13-V5 plus EV, (*D*) N400Q/N440Q-TMPRSS13-V5 plus EV, (*E*) N400Q/N440Q-TMPRSS13-V5 plus HAI-2-EYFP, or (*F*) EV plus EV. Cells were fixed, permeabilized, and incubated overnight with anti-V5 to detect TMPRSS13 or anti-KDEL to detect endogenous KDEL. Nuclei (DAPI) (*blue, A-F*), TMPRSS13-V5 (*red, A-F*), HAI-2 (*green, B and E*), KDEL (*cyan, A-F*). Merged images of TMPRSS13/KDEL are shown in panels on the right. Yellow boxes indicate the regions of the images that were cropped for Figure 4.

**Figure S7**. *A*, HEK293T cells transfected with WT-TMPRSS13 (T13) + empty vector (EV), WT-TMPRSS13+HAI-2, N400Q/N440Q-TMPRSS13+EV, or N400Q/N440Q+HAI-2 were immunoprecipitated with anti-V5 antibody and separated by SDS-PAGE under reducing conditions using a 10% gel. Whole cell lysates were included to verify protein expression prior to immunoprecipitation (*input*). Proteins were detected by western blotting using anti-V5 and anti-HAI-2 antibodies. *B*, Quantification of HAI-2 interaction with WT-TMPRSS13-V5 or N400Q/N440Q-TMPRSS13-V5, normalized to V5 expression. Error bar is representative of

standard deviation. Student’s t-test was used to evaluate the difference in TMPRSS13/HAI-2 interaction. Results for four biological replicates are shown.

# Supplementary Figure 1

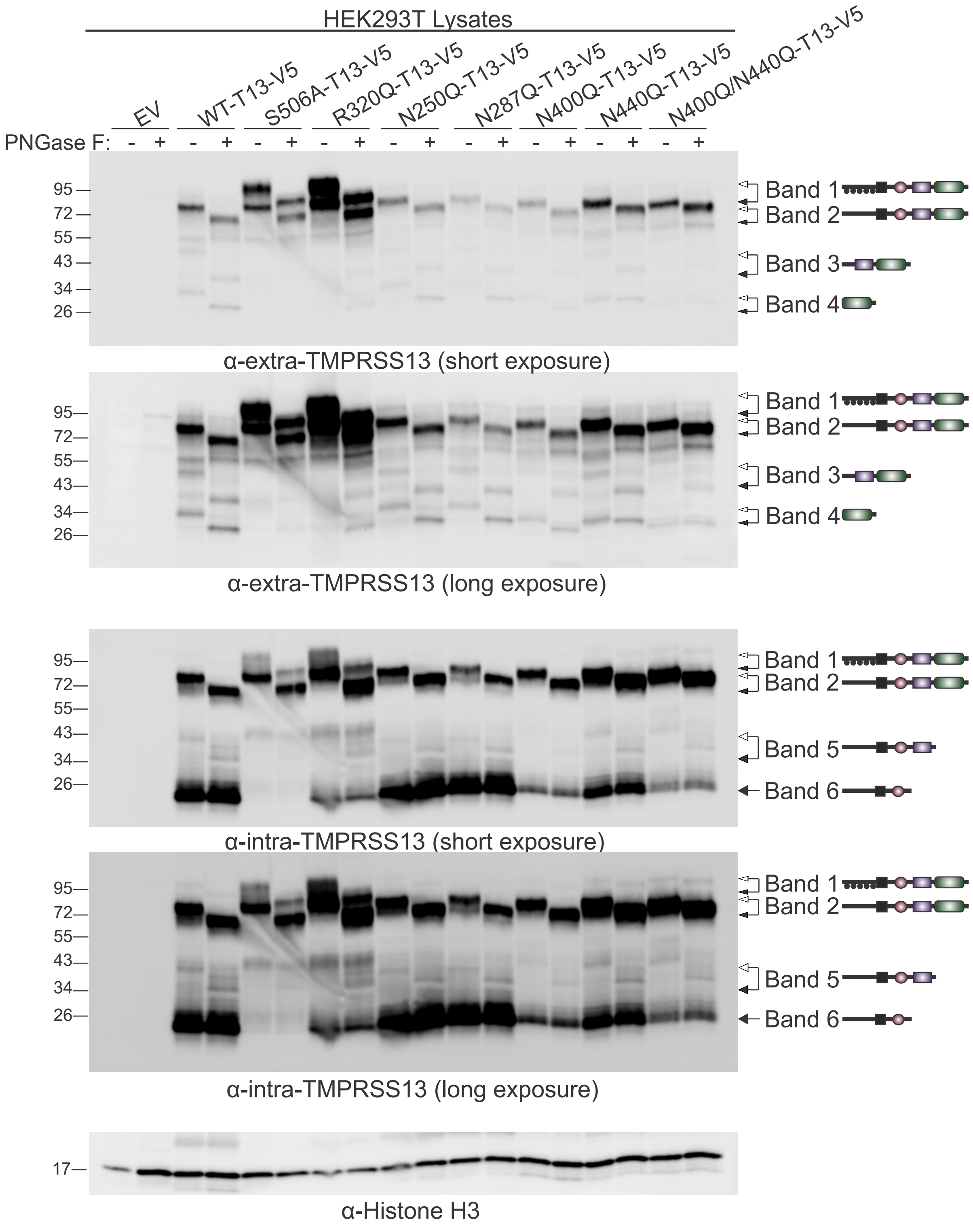

# Supplementary Figure 2

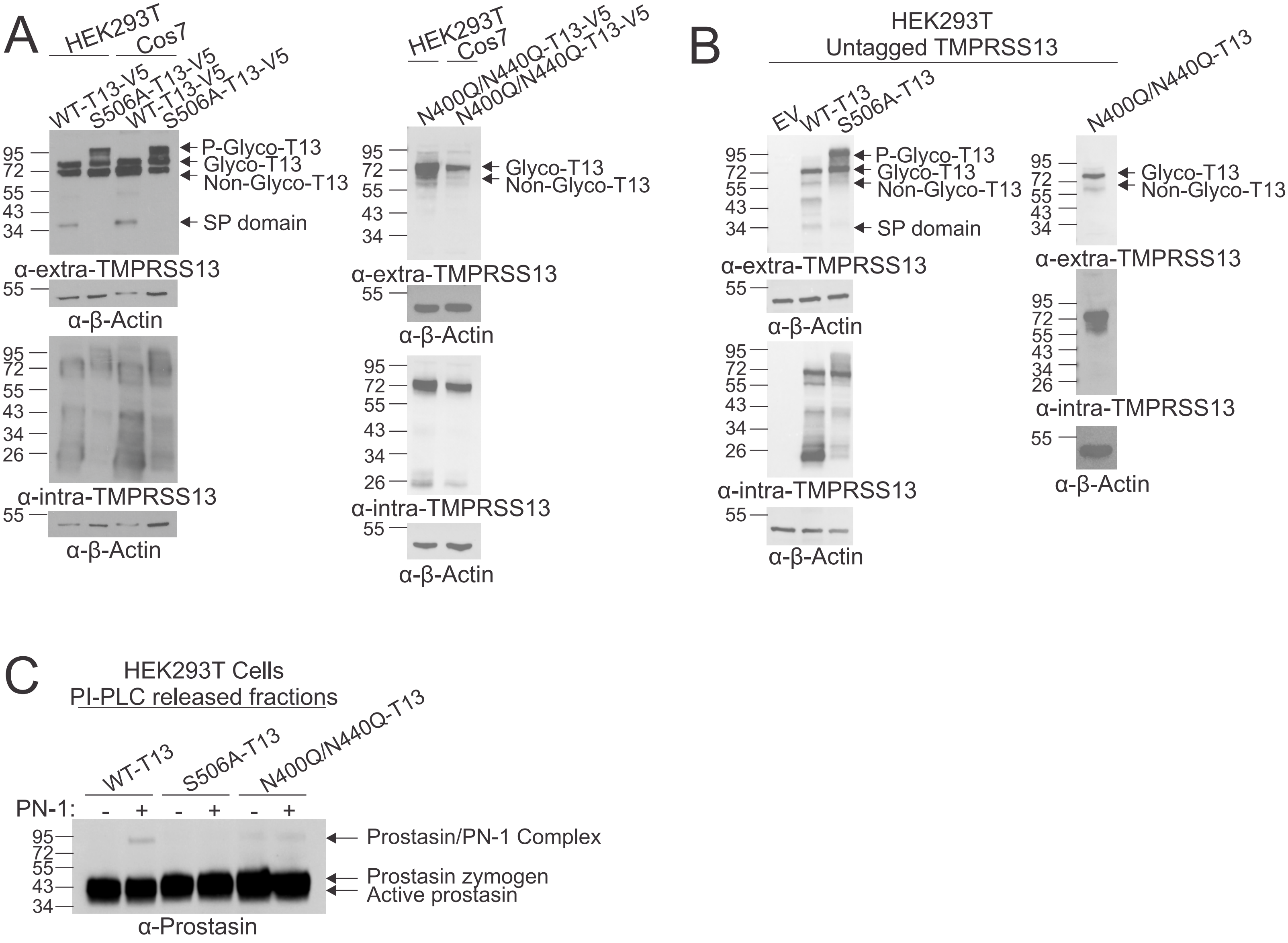

# Supplementary Figure 3

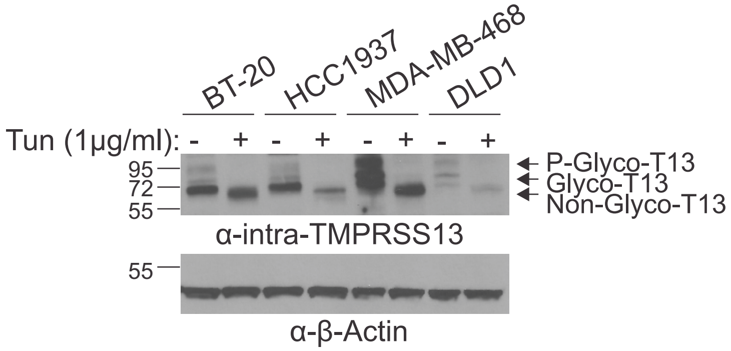

Supplementary Figure 4

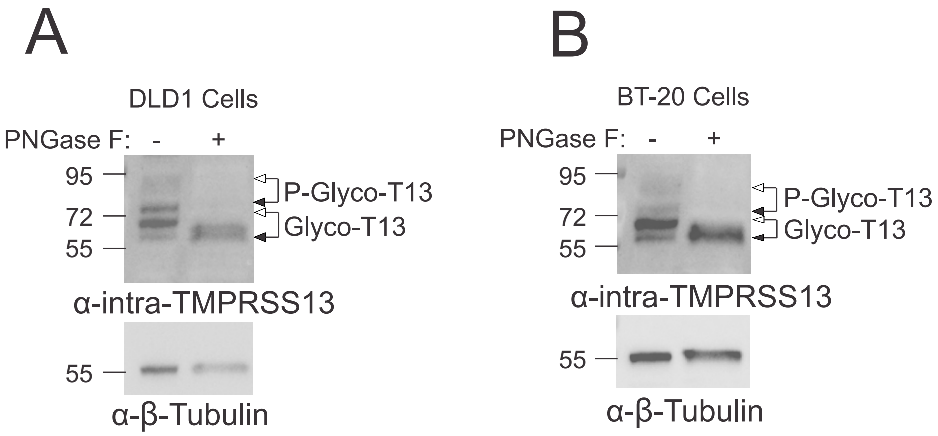

# Supplementary Figure 5

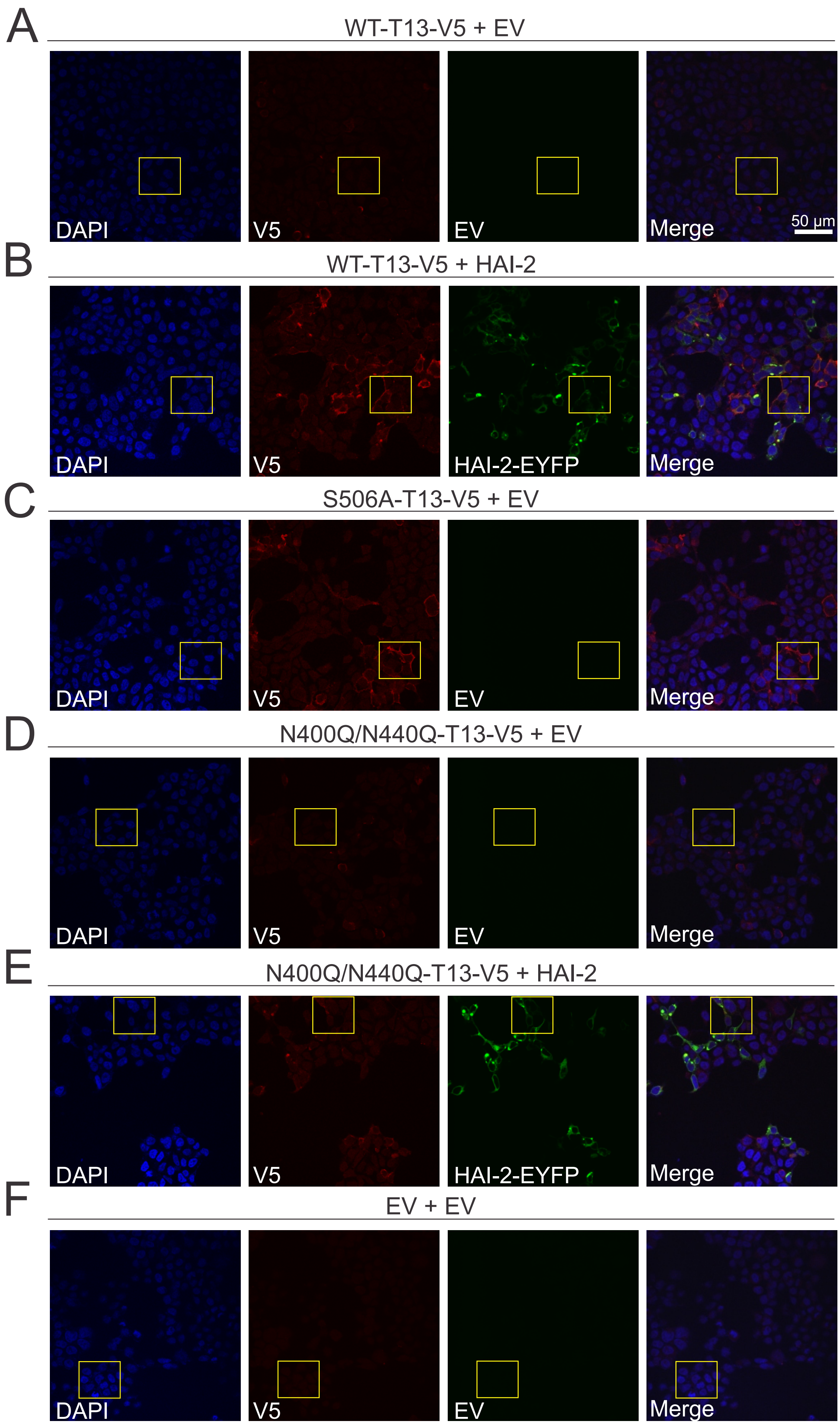

Supplementary Figure 6

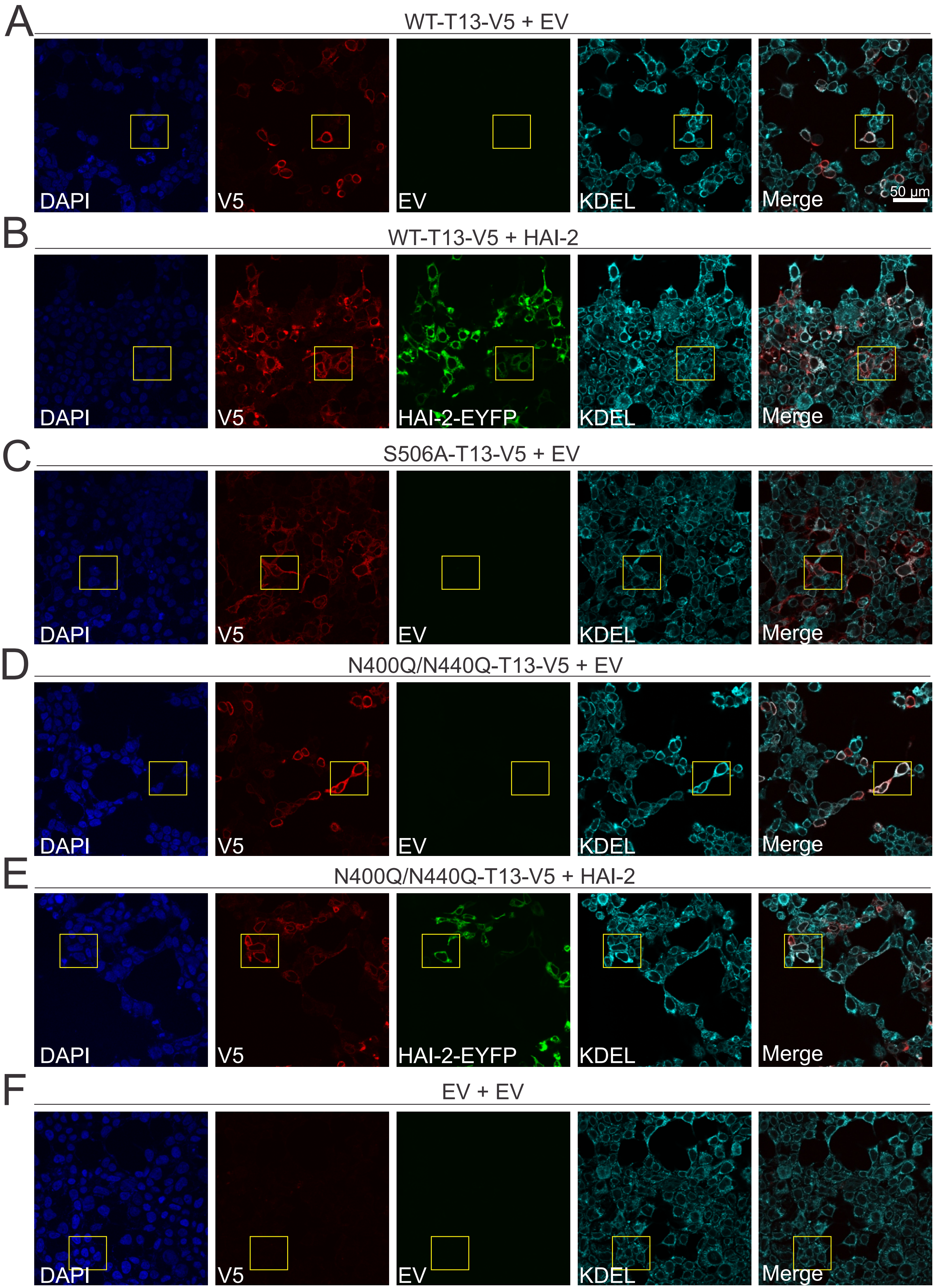

# Supplementary Figure 7

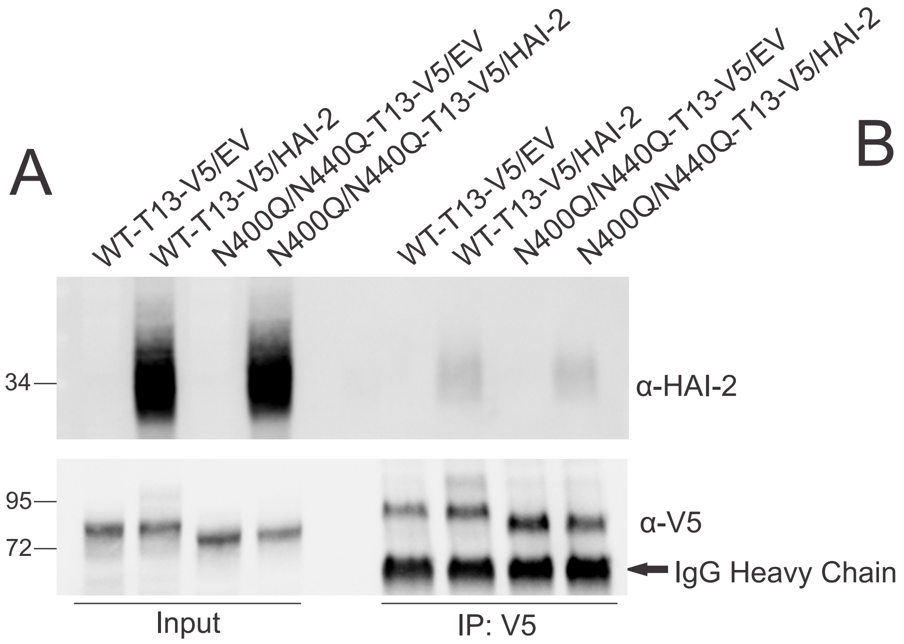


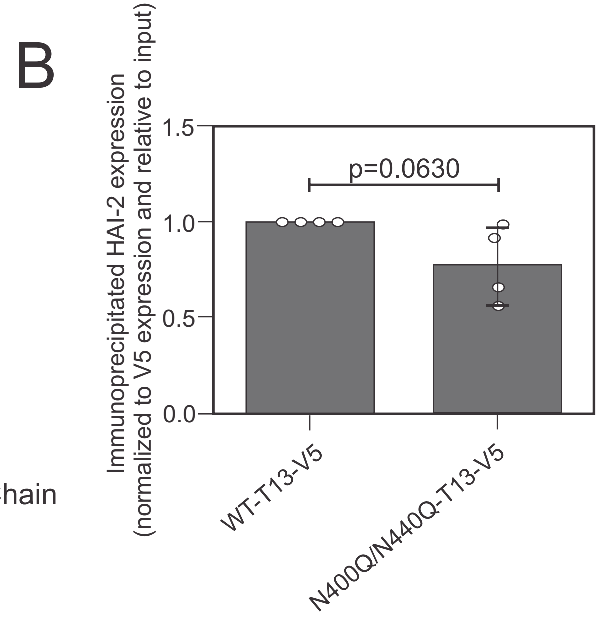

Supplement: Supplemental Figures S1–S7 [file mmc1.pdf]
